# Relationship between community walking ability and in-hospital mortality in elderly patients with sepsis: a single-center retrospective cohort study

**DOI:** 10.1186/s40560-019-0385-1

**Published:** 2019-05-21

**Authors:** Ryo Ueno, Atsushi Shiraishi, Ryohei Yamamoto, Seibi Kobara, Yoshiro Hayashi

**Affiliations:** 1 Department of Intensive Care Medicine, Kameda Medical Center, 929, Higashi-cho, Kamogawa, Chiba 296-0041 Japan; 2 Emergency and Trauma Center, Kameda Medical Center, 929, Higashi-cho, Kamogawa, Chiba 296-0041 Japan; 3 Department of Rehabilitation, Kameda Medical Center, 929, Higashi-cho, Kamogawa, Chiba 296-0041 Japan

**Keywords:** Adults, Frailty, Mortality, Sepsis

## Abstract

**Purpose:**

To examine the association of a simple frailty assessment, Life Space (LS), with in-hospital mortality in elderly patients with sepsis.

**Methods:**

We used data from a single hospital between 2014 and 2017. We included elderly patients (age ≥ 65 years) admitted to the intensive care unit (ICU) with sepsis, as defined by sepsis-3 criteria. Frailty assessment was based on a patient’s ability to independently go out of the house before the ICU admission. We termed this dichotomous score as Life Space. The primary outcome was in-hospital mortality. Logistic regression was used to investigate the association of LS with each outcome after adjusting for age, sex, and Sequential Organ Failure Assessment score.

**Results:**

Of the 335 participants included in the final analysis, 121 (36%) were classified as frail. LS-positive patients had a higher in-hospital mortality (adjusted odds ratio (aOR) 2.32; 95% confidence interval (CI) 1.36–3.96; *P* = 0.002) than did LS-negative patients. We observed similar patterns in six sets of sensitivity analyses after accounting for different confounders. In subgroup analyses, significant interactions were observed in participants with versus those without treatment limitations (aOR 1.02 vs. 2.66, *P* for interaction = 0.042).

**Conclusions:**

In this single-center study, frailty assessed by LS was independently associated with a higher in-hospital mortality.

**Electronic supplementary material:**

The online version of this article (10.1186/s40560-019-0385-1) contains supplementary material, which is available to authorized users.

## Background

Sepsis is a global burden, especially in older adults, because of its high mortality and morbidity [1]. There is increasing evidence that a patient’s health status before the onset of sepsis plays a pivotal role in the progression and sequelae of sepsis [2–6]. Frailty, which is theoretically defined as a geriatric multidimensional syndrome that is assessed by one’s physiological function rather than chronological age, is one of the indices that represent a patient’s ability to recover from an episode of acute illness.

There are, however, significant barriers to the assessment of frailty in the intensive care unit (ICU). Most of the traditional scores including Frailty Index [7], Clinical Frailty Scale (CFS) [8], and Life Space Assessment [9, 10] require additional manual processes [7], which are prone to inter-rater errors and, sometimes, are time-consuming. Automated estimation of frailty was recently suggested [11]; however, it requires the implementation of administrative steps such as coding of the diagnosis, which cannot be immediately implemented in acute settings. Despite the clinical importance of bedside frailty assessment, there is a dearth of research towards the development and validation of a quick frailty assessment tool.

In this context, we focused on a simplified frailty assessment framework based on a patient’s ability to independently go out of the house and venture into the community. We termed this dichotomous score as Life Space (LS). We hypothesized a priori that LS is an independent risk factor of hospital mortality and conducted this single-center retrospective cohort study to investigate the association of LS with in-hospital mortality.

## Methods

### Study design

We conducted this single-center retrospective cohort study in a closed mixed-ICU system in a tertiary teaching hospital in a rural area in Japan, where the population aging rate (age ≥ 65 years) was > 30%. This study was reviewed and approved by the Kameda Medical Center’s Institutional Review Board. The committee waived the requirement for informed consent for all subjects enrolled in this study due to the retrospective design of the study. This study was conducted in accordance with the STROBE guidelines [12] for reporting.

### Study population

All consecutive patients aged 65 years or older and admitted to the ICU between September 2014 and January 2017 with a diagnosis of sepsis, which was retrospectively confirmed by trained intensive care physicians using sepsis-3 criteria [13], were included. Patients who developed sepsis after the ICU admission were excluded. We excluded patients who underwent elective surgeries or stayed in the ICU for < 24 h because it was unlikely that the frailties of those patients were assessed in the ICU by physiotherapists.

### Data collection

We collected the following data: age, sex, admission category (medical or emergency surgery), septic shock (defined by sepsis-3 criteria [13]), previous ICU admission, Charlson Comorbidity Index [14], treatment limitations (limitations in providing ICU-specific life-sustaining therapies such as cardiopulmonary resuscitation, mechanical ventilation, and vasopressors or renal replacement therapy), and the site of infection (abdominal, respiratory, urinary, and others). The severity of a patient’s status was assessed using the Acute Physiology and Chronic Health Evaluation (APACHE) II score [15], Simplified Acute Physiology Score (SAPS) II [16], and Sequential Organ Failure Assessment (SOFA) score [17]. The SOFA score was manually calculated at the time of ICU admission. We also collected data regarding the use of mechanical ventilation and administration of noradrenaline and/or corticosteroids.

### Frailty assessment

**Fig. 1:**
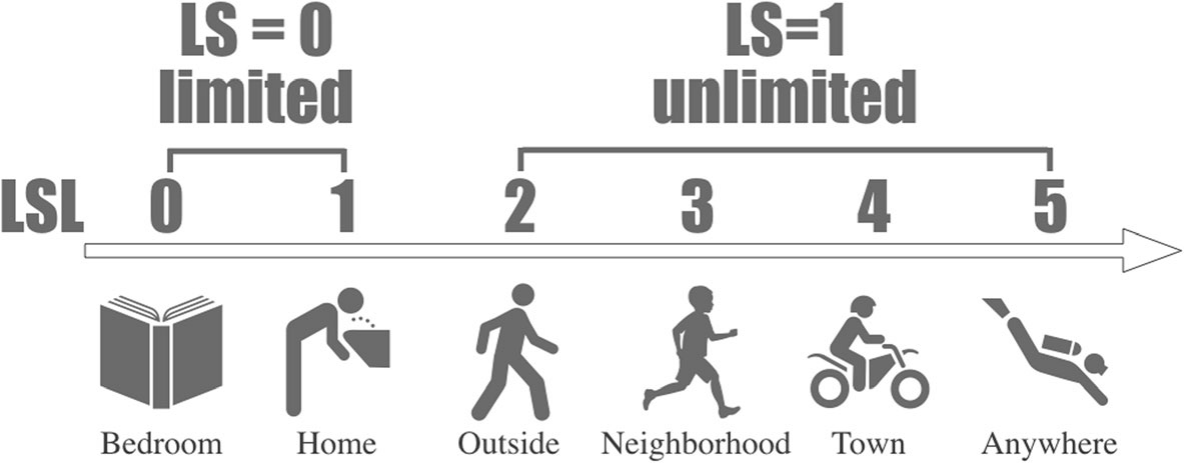
Life Space (LS). Life Space Level (LSL) was scored by asking a patient how far could he/she move independently without limitations before the onset of symptoms of critical illnesses, ranging from his/her bedroom (score = 0) to one’s town (score = 5). We retrospectively collected LSL scores and categorized patients into two groups: those who could not go out of their houses (LSL = 0 or 1) were categorized as the “limited” group (LS = 0), while all others (LSL ≥ 2) were classified as the “unlimited” group (LS = 1)

Frailty was initially assessed using Life Space Level (LSL). LSL is a component of the Life Space Assessment Score [10], which is widely used in the functional assessment of elderly patients [18–20]. LSL was scored by asking a patient how far he/she could move independently without limitations before the onset of the symptoms of critical illnesses, and it ranged from one’s bedroom (score = 0) to one’s town (score = 5). Physiotherapists with at least 5 years of clinical experience collected it on the first day of rehabilitation, which is usually within 24–48 h of ICU admission. If a patient was unable to provide this information, LSL was estimated on the basis of an interview with his/her family. We retrospectively collected these LSL scores from the physiotherapy electronic health records and categorized the patients into two groups: those who cannot go out of their houses (LSL = 0 or 1) were categorized in the “limited” group, while all others were categorized in the “unlimited” group (LSL ≥ 2). We named this dichotomous score as Life Space (LS) (Fig. 1).

### Outcome measurement

The primary outcome was in-hospital mortality. Secondary outcomes included 28-day and 90-day mortalities. All patients were followed up by the respective ICU doctors after 3 months of the ICU admission by electronic health record review.

### Statistical methods

We compared the characteristics of patients in the limited and unlimited groups using the chi-square or Wilcoxon signed rank test, as appropriate. A logistic regression model adjusted for age, sex, and SOFA score on admission to the ICU was used to investigate the association between LS and each outcome. A set of potential confounders was chosen a priori based on the clinical plausibility and previous studies [4, 5, 13, 21]. LS was unlikely to be measured in severely sick patients since they were unlikely to be assessed by physiotherapists; therefore, we assumed that these study variables were missing at random [22]. We imputed the missing values using the R package “mice” [23]. A hundred datasets were created after 50 iterations for each value. Point and interval estimates were combined using Rubin’s rule [22].

Sensitivity analyses included several logistic regression models to assess the robustness of the primary analysis. The models were adjusted as follows: model 1 for age, sex, LS, SOFA score, and Charlson Comorbidity Index; model 2 for age, sex, LS, SOFA score, and admission category; model 3 for age, sex, LS, SOFA score, Charlson Comorbidity Index, and admission category; model 4 for age, sex, LS, and APACHE II score; and model 5 for age, sex, LS, and SAPS II. Next, we repeated the analyses with generalized estimating equations (GEE) in order to account for the potential clustering of cases of sepsis within each source of infection. Additionally, to assess the heterogeneity of the different levels of treatment, we conducted subgroup analysis of patients with and without treatment limitations and patients with age more than, equal to, or less than 80 years. Finally, we conducted a complete case analysis to ensure the robustness of the multiple imputations. Categorical variables were expressed as percentages, whereas continuous variables were described as means (± standard deviations (SDs)). A two-sided *P* value less than 0.05 was considered to indicate statistical significance. The analyses were performed using R software, version 3.3.2 (The R Foundation for Statistical Computing, Vienna, Austria).

## Results

### Patient characteristics

**Fig. 2:**
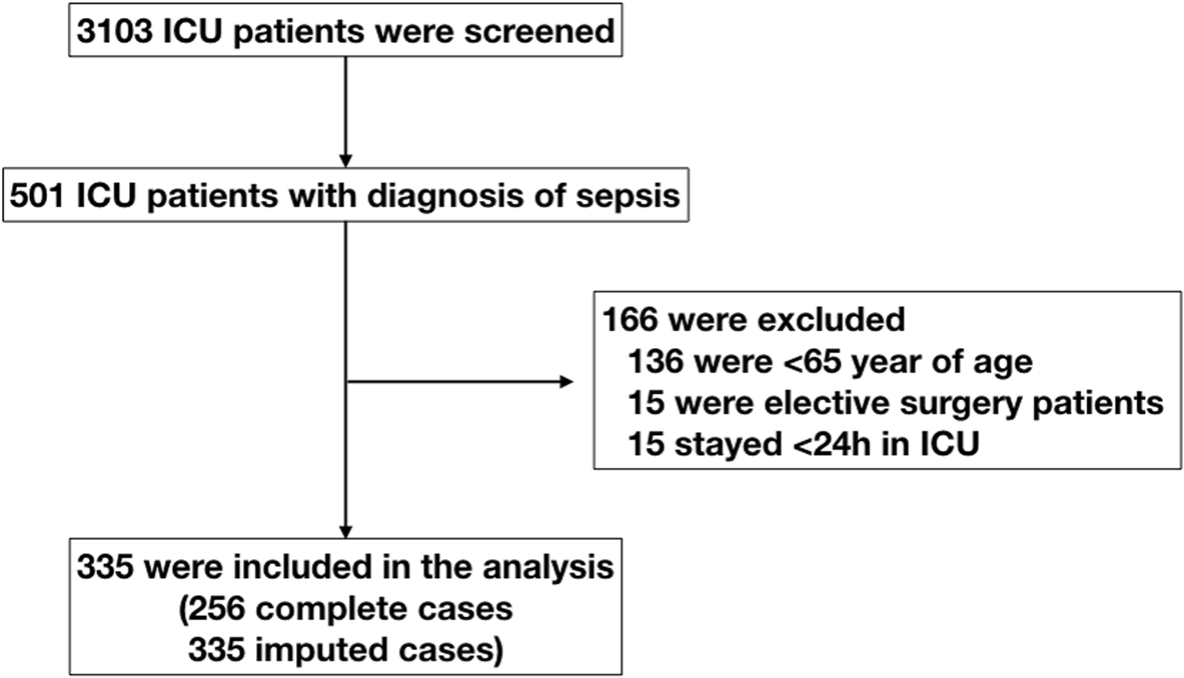
Number of patients in the intensive care unit (ICU) screened and included in primary analysis

Overall, 3103 patients were admitted to the intensive care unit within the study period; 501 patients with a diagnosis of sepsis were included in this study. Of these, 135 were excluded because their age was < 65 years, 15 were excluded because of elective surgeries, and 15 were excluded because their duration of ICU stay was < 24 h. Finally, 335 patients were included in the analyses (Fig. 2). Of these, 121 patients were categorized in the limited group based on LS. Compared with the unlimited group, the patients in the limited group were significantly older (80 vs. 77 years, *P* < 0.001) and had higher APACHE II score (23 vs. 21, *p* = 0.043), more frequent use of vasopressors (72.7% vs. 61.2%, *P* = 0.045) and corticosteroids (37.2% vs. 19.2%, *P* < 0.001), and more frequent treatment limitations (i.e., do-not-resuscitate, do-not-dialyze, and do-not-intubate) (32.2% vs. 14.5%, *P* < 0.001). These patients with limited function had higher in-hospital mortality (45.5% vs. 24.8%, *P* < 0.001), 28-day mortality (35.5% vs. 15.4%, *P* < 0.001), and 90-day mortality (47.9% vs. 24.8*%, P* < 0.001). Other patient characteristics are summarized in Table 1. Our dataset had the following missing values: LS in 79 patients, 90-day mortality in 35 patients, and 28-day mortality in 5 patients.

**Table 1:** Baseline characteristics of elderly patients in the intensive care unit (ICU) with sepsis

Variable		Limited group (*N* = 121)	Unlimited group (*N* = 214)	*P* value
Demographics	Age, years (mean [SD])	80 (6.90)	77 (7.32)	< 0.001
	Male sex, *n* (%)	75 (62.0)	150 (70.1)	0.162
	Admission category, *n* (%)			1
	Medical	93 (76.9)	164 (76.6)	
	Emergency surgery	28 (23.1)	50 (23.4)	
	Septic shock, *n* (%)	81 (66.9)	116 (54.2)	0.031
	Previous ICU admission, *n* (%)	8 (6.6)	21 (9.8)	0.424
	APACHE II score (mean [SD])	23 (8.79)	21 (7.89)	0.043
	SAPS II (mean [SD])	53 (18.44)	50 (16.0)	0.094
	SOFA score (mean [SD])	9 (3.56)	8 (3.73)	0.081
	Charlson Comorbidity Index (mean [SD])	3 (2.24)	2 (2.00)	0.082
	Treatment limitation*, *n* (%)	39 (32.2)	31 (14.5)	< 0.001
	Site of infection, *n* (%)			0.027
	Abdominal	35 (28.9)	68 (31.8)	
	Respiratory	36 (29.8)	68 (31.8)	
	Urinary	30 (24.8)	30 (14.0)	
	Others	9 (7.4)	35 (16.4)	
	Unknown	11 (9.1)	13 (6.1)	
	FIM (mean [SD])	37 (22.6)	55 (28.8)	< 0.001
	Barthel Index (mean [SD])	12 (19.8)	25 (29.7)	< 0.001
Interventions	Mechanical ventilation, *n* (%)	53 (43.8)	93 (43.5)	1
	Noradrenaline use, *n* (%)	88 (72.7)	131 (61.2)	0.045
	Corticosteroid use, *n* (%)	45 (37.2)	41 (19.2)	< 0.001

*SD* standard deviation, *APACHE* Acute Physiology and Chronic Health Evaluation, *SAPS* Simplified Acute Physiology Score, *SOFA* Sequential Organ Failure Assessment, *FIM* Functional Independence Measure

^*^Limitation in the provision of ICU-specific life-sustaining therapies (e.g., cardiopulmonary resuscitation, mechanical ventilation, use of vasopressors, and renal replacement therapy) documented in the medical records

### Association between frailty and mortality in elderly adults

Table 2 summarizes the adjusted associations between LS and each outcome. LS was associated with higher in-hospital mortality after adjustment for age, sex, and SOFA score (adjusted odds ratio (aOR) 2.32; 95% confidence interval (CI) 1.36–3.96; *P* = 0.002). This finding was consistent in the secondary outcomes as well; LS was an independent risk factor of 28-day mortality (aOR 3.47; 95% CI 1.87–6.46; *P* < 0.001) and 90-day mortality (aOR 2.56; 95% CI 1.46–4.47; *P* = 0.002).

**Table 2:** Multivariate analysis of Life Space with primary and secondary outcomes

	Adjusted OR (95% CI)	*P* value
In-hospital mortality	2.32 (1.36–3.96)	0.002
28-day mortality	3.47 (1.87–6.46)	< 0.001
90-day mortality	2.56 (1.46–4.47)	0.001

Adjusted for age, sex, and SOFA score

*OR* odds ratio, *CI* confidence interval, *SOFA* Sequential Organ Failure Assessment

### Sensitivity analysis

**Fig. 3:**
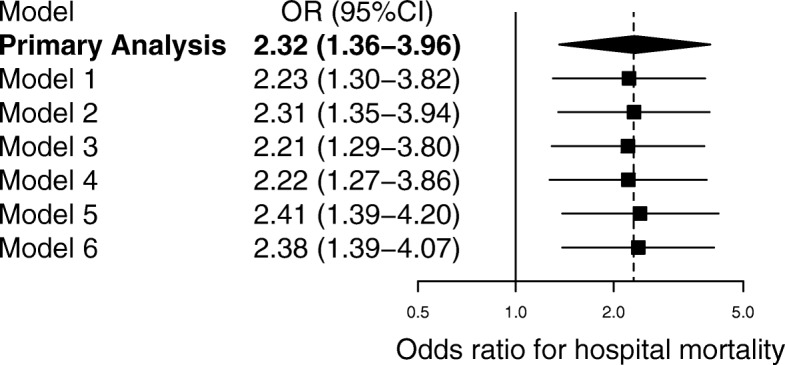
Sensitivity analyses for potential confounders. Primary analysis was further adjusted for each potential confounder. Odds ratios greater than 1.0 indicate an increased risk of death. The models were adjusted as follows: primary analysis for age, sex, and Sequential Organ Failure Assessment (SOFA) score; model 1 for age, sex, SOFA score, and Charlson Comorbidity Index; model 2 for age, sex, SOFA score, and admission category (medical or surgical); model 3 for age, sex, SOFA score, Charlson Comorbidity Index, and admission category; model 4 for age, sex, and Acute Physiology and Chronic Health Evaluation (APACHE) II score; model 5 for age, sex, and Simplified Acute Physiology Score (SAPS) II; and model 6 for age, sex, and SOFA score using generalized estimating equations (GEE) with each source of infection. Estimates are shown as mean differences with 95% confidence intervals

**Fig. 4:**
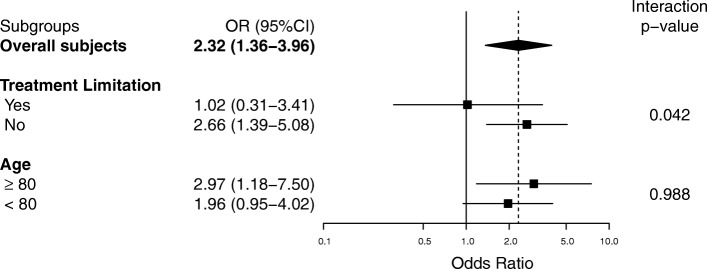
Adjusted odds ratios for in-hospital mortality in subgroups of patients with sepsis. In the subgroups of patients, odds ratios are indicated by solid squares. Horizontal lines represent 95% confidence intervals (95% CI). Odds ratios greater than 1.0 indicate an increased risk of in-hospital death

In the sensitivity analysis, the association between LS and in-hospital mortality remained similar in various multivariate analyses (Fig. 3). In the complete case analysis, however, we found no significant associations between LS and in-hospital mortality (aOR 1.43; 95% CI 0.76–2.69; *P* = 0.267). In addition, supplemental analyses to assess association of LS and the study outcome were shown (Additional file 1: Table S1 and S2). In the subgroup analysis, significant interactions were observed between participants with treatment limitations (aOR 1.02; 95% CI 0.31–3.41) and those without (aOR 2.66; 95% CI 1.39–5.08) (*P* = 0.042). No significant interactions were observed in participants divided on the basis of age (Fig. 4).

## Discussion

In this retrospective study, we investigated 335 elderly ICU patients with sepsis to investigate the association between in-hospital mortality and LS. Multivariate analysis identified LS as an independent risk factor of in-hospital mortality. This association was consistent in multiple sensitivity analyses with different statistical assumptions. To the best of our knowledge, this is the first investigation to develop and evaluate such a straightforward assessment of frailty in patients with sepsis in ICU settings.

Recently, there has been a growing understanding that frailty may be a more robust predictor of vulnerability than chronological age alone [24]. Several studies have reported that frailty is associated with both poor short-term [2, 5, 25–27] and long-term [3, 4, 28–30] outcomes in patients in the ICU. Therefore, several statements support the use of frailty in the triage [31–36], as an entrance to the ICU, while making decisions with respect to treatment limitations [37], as an exit from the ICU. Though there is a growing demand for bedside assessment of frailty [24], only a few scores have been developed for such acute care settings. Even the most widely investigated scores, such as Frailty Index [7] and CFS [8], require additional manual steps and training, which could be substantial hurdles against their implementation in acute settings. In this context, several studies have investigated the utility of quick bedside assessments of frailty such as handgrip strength (HGS) [38], mid-arm circumference [39], and quadriceps muscle thickness [40]. Of these, HGS is one of the most widely investigated assessment tools. One multicenter prospective cohort study reported a strong positive association between HGS and in-hospital mortality in intubated patients in the ICU [38]. However, HGS is different from other frailty assessment tools in that it measures the physiological weakness during the ICU admission rather than that before the onset of critical illness. Additionally, there is no standardized protocol for the measurement of HGS, which obstructs the integration of multiple evidences into practice [41]. Overall, we are yet to find the ideal bedside assessment test of frailty in the ICU.

The advantages of LS are its simplicity and objectivity, which are essential in clinical use. Additionally, consistent with the results of the aforementioned trials with other frailty assessment tools [2, 5, 25–27, 38], LS was an independent risk factor of in-hospital mortality in our analysis. Of note, we also found that the association between frailty and mortality was influenced by the presence of treatment limitations, i.e., patients with treatment limitations had high aOR of death regardless of their frailty status. We can posit that this interaction might be a result of the different treatment approaches; however, our data and analyses were not conclusive enough to prove it. Further studies are needed to validate our findings.

## Limitations

Our study had several potential limitations. First, LS data were not available in approximately a quarter of our patients. An interview with the physiotherapists involved in this study revealed that LS was missing in the following three types of patients: (1) critically unstable patients without rehabilitation orders, (2) transferred patients without rehabilitation orders, and (3) unconscious or agitated patients without family members available. This observation supports a systematic relationship between the propensity of missing values and the observed severity data (missing at random); therefore, we supported the results of our first imputation using mice rather than the results of complete case analysis, which assumes no relationship between the missing data and observed data (missing completely at random). Our results were not robust enough to conclusively affirm our hypothesis; however, they were persuasive enough to facilitate further investigation into the prospective assessment of LS.

Second, the inter-rater reliability of LS was not completely confirmed. Theoretically, an inter-rater error is possible in our dataset because we did not comprehensively define LS when the physiotherapists collected the data. Clinically, however, the probability of such an error is small. Whether one can individually go out of the house is a simple and objective question. We decided a priori to avoid using the six categories of functional assessment of LS for the sake of better inter-rater reliability. These approaches minimized the likelihood of such an error.

Third, the results of this single-center study could be prone to information bias, i.e., an unblinded association between the index test (LS) and the study outcome. However, LS was measured within 48 h of ICU admission, independent of the outcome measurement; therefore, any interaction between LS and the outcomes was minimized.

Forth, we could not directly compare LS with other frailty measurements such as Frailty Index and CFS.

Finally, we could not measure the long-term outcomes in patients with limited function. Recent studies [28, 29] have reported a strong correlation between pre-hospital frailty and long-term outcomes such as cognitive function and mortality. LS should also be assessed in this context in future trials.

## Conclusion

In this single-center study that included 335 elderly adults with sepsis, LS was associated with in-hospital mortality. This association persisted across the sensitivity analyses with multiple statistical assumptions. Our findings can be utilized in the development of a quick frailty assessment tool.

## Additional file

Additional file 1: **Table S1.** Prediction ability of the reference and LS models for in-hospital mortality. Table S2 Propensity-match analysis* of Life Space with primary and secondary outcomes. (DOCX 15 kb)

## Abbreviations

APACHE II score: Acute Physiology and Chronic Health Evaluation II score
CFS: Clinical Frailty Scale
CI: Confidence interval
GEE: Generalized estimating equations
HGS: Hand grip strength
ICU: Intensive care unit
LS: Life Space
LSL: Life Space Level
OR: Odds ratio
SAPS II: Simplified Acute Physiology Score II
SD: Standard deviations
SOFA: Sequential Organ Failure Assessment
