# Supplementary material for: Relationship between community walking ability and in-hospital mortality in elderly patients with sepsis: a single-center retrospective cohort study
Source: J Intensive Care. 2019 May 21;7:33. doi: 10.1186/s40560-019-0385-1 (PMC6528228; doi:10.1186/s40560-019-0385-1)
Supplement: Supplementary file 1 — Table S1. Prediction ability of the reference and LS models for in-hospital mortality. Table S2 Propensity-match analysis* of Life Space with primary and secondary outcomes. (DOCX 15 kb) [file 40560_2019_385_MOESM1_ESM.docx]

Additional file 1

**Table S1** Prediction ability of the reference and LS models for in-hospital mortality

| **Outcome** | **C-statistic** | **P value*** |
| --- | --- | --- |
| Reference model (age, sex, and SOFA score) | 0.67 (0.60–0.73) | Reference |
| LS model (age, sex, SOFA score, and LS) | 0.71 (0.65–0.77) | 0.03 |

*Comparison of C-statistics of the reference model (logistic regression with age, sex, and SOFA score) with that of LS model (logistic regression with age, sex, SOFA score, and LS) using DeLong’s test.

**Table S2** Propensity-match analysis* of Life Space with primary and secondary outcomes

|  | Adjusted OR (95% CI) | p-value |
| --- | --- | --- |
| In-hospital mortality | 2.19 (1.17–4.09) | 0.014 |
| 28-day mortality | 3.33 (1.57–7.05) | 0.002 |
| 90-day mortality | 2.47 (1.30–4.67) | 0.006 |

*Adjusted for age, sex, and SOFA score

OR, odds ratio; CI, confidence interval; SOFA, Sequential Organ Failure Assessment.
